# Oral Hygiene, Report of Committee on Oral Hygiene of National Dental Association

**Published:** 1909-01-15

**Authors:** 


### ORAL HYGIENE.

Report of Committee on Oral Hygiene of National Dental Association for the Education of the Public in the Care of the Mouth and Teeth.

A HEALTHY mouth and sound teeth are essential to the general well-being. Many diseases of the body cause unhealthy conditions of the mouth and teeth, and on the other hand, diseases of the mouth and teeth are prejudicial to the general health.

The value of a good set of teeth can hardly be over-estimated, for mastication is the first step in a series of processes by which the food is transformed into nourishment adapted to the needs of the system. The teeth are also important aids in forming speech and in maintaining the symmetry of the features. For these reasons all causes promoting destruction of the teeth should be avoided.

Decay is not the only enemy of the teeth; inflammation and absorption of the gums and sockets, chiefly caused by the presence of tartar, is alarmingly frequent, and results in the loss of very many teeth.

To prevent decay of the teeth as well as the destruction of their sockets, the one great essential is *cleanliness of the mouth.*

*Are the temporary teeth of much importance?*

They are, and should be cared for from their first appearance until they are displaced by their permanent successors. A child needs teeth for mastication as much as does an adult. If the first teeth are decayed, chewing is painful and is avoided and the habit of swallowing food unchewed becomes established. The results of this practice are indigestion and many stomach and bowel disorders. If decayed and painful first teeth are prematurely extracted, the result is generally irregularity and crowding of the second set, they being thus made unsightly, inefficient for chewing, and more liable to decay.

*How long should the second set of teeth last?*

To the end of life.

*How do we lose teeth?*

Chiefly by decay and loosening.

*What causes teeth to decay?*

Decay is caused by the fermentation of the remains of food clinging between the teeth and in their depressions, with the production of an acid.

*Can decay be prevented?*

Yes, to a large extent.

*How can decay be prevented?*

By scrubbing the teeth thoroughly with a tooth brush, tooth-powder, and water, and by the preservation of the general health.

*What causes teeth to loosen?*

Chiefly the presence of tartar.

*How can the loosening of teeth be prevented?*

By preventing the accumulation of tartar or other irritants which inflame the gum around the tooth, by scrubbing with brush and powder, the same as for the prevention of decay. Tartar which cannot be reached by the brush should be removed by the dentist.

*How often should the teeth be cleaned?*

At least twice a day; in the morning and at bedtime; better after each meal. The after-meal cleansing should not involve a too vigorous use of the brush.

*What kind of a tooth-powder should be used?*

For purposes of simple cleanliness in a fairly healthy mouth, the best tooth-powder is made of some alkaline substance such as precipitated chalk or magnesia so finely powdered as to be without perceptible grit, and flavored to taste as desired, with aromatic oil such as oil of wintergreen, oil of peppermint, etc. The powder should not contain orris-root or other starchy vegetable matter. In cases where the gums or teeth, or both, are much diseased, special powders or lotions are needed; these should be prescribed by the dentist.

*How often should the tooth-powder be used?*

At least once a day; preferably at bedtime.

*How should the tooth-brush be used?*

Skill and care, and not force, are required to secure the best results. The most effective routine is to first rinse the mouth with tepid water to wash away particles of food; next use the brush without powder to dislodge any particles remaining about the teeth, rinse again with water, and lastly, use the brush with powder to better scour the teeth. By its alkalinity the powder helps also to neutralize the acids causing tooth-decay. The usual crosswise brushing often forces food into the spaces between the teeth, where it does the most harm. To remove it, the brush should have bristles well apart and in uneven lengths. For the inner and outer surfaces, the teeth should be brushed downward and upward; for the grinding surfaces, backward and forward and from side to side; in this way all parts of the teeth can be cleansed from foreign matter. It should be kept in mind that the surfaces most difficult to reach are the ones requiring the greatest care. This very thorough brushing is required only once a day. It must be remembered, however, that some mouths and teeth are more subject to disease than others, and require greater care.

The use of a quill toothpick and waxed floss silk will remove food particles that cannot be dislodged with the brush. In using the silk, care must be taken not to unduly force the silk upon the gum and injure its union with the neck of the tooth. A similar injury can be caused by the improper use of the toothpick.

*How often should the dentist be visited?*

At least twice a year; when unusual conditions predisposing to decay and gum trouble are present, the visits should be made more frequently.

For the cure of unhealthy conditions resulting in a predisposition to decay of the teeth and diseases of the gums, systematic as well as local treatment is often necessary. Usually the treatment found most effective is that which promotes the general health of the patient, and includes nutritious food, sunshine, exercise, and sleep.—*Cosmos.*

